# Evaluation of the accuracy of full-arch impressions between three different intraoral scanners and conventional impressions: A prospective in vivo study

**DOI:** 10.34172/joddd.40841

**Published:** 2024-03-29

**Authors:** Niharika Bhatia, Srirengalakshmi Muthuswamy Pandian

**Affiliations:** Department of Orthodontics, Saveetha Dental College and Hospital, Chennai, 600077, India

**Keywords:** Dental, Impression material, Orthodontics

## Abstract

**Background.:**

This in-vivo study evaluated the accuracy of full-arch digital impressions obtained through different intraoral scanning technologies regarding trueness and efficiency against the standard alginate impressions.

**Methods.:**

Alginate impressions were taken from 50 subjects, and the resulting stone casts were scanned using the Trios 3Shape desktop scanner. In-vivo scans were conducted on each participant using three intraoral scanners: Medit, CEREC Primescan, and 3Shape Trios. The scanned files were superimposed onto two software platforms: the 3Shape Orthoanalyser and Geomagic software. This superimposition was performed against the reference model to calculate 3D and 2D deviations, enabling efficiency comparisons between digital and traditional workflows based on work time in minutes. Measurements and comparisons were made in three planes: transverse, sagittal, and vertical dimensions for all the models and stone casts. Statistical analysis employed SPSS 23, with the significance level set at *P*<0.05.

**Results.:**

Significant deviations were observed between the three intraoral scanners and the alginate impression, with molar and premolar areas showing greater imprecision across dental arches. Compared to the alginate technique, Medit i500 tended to reduce the transverse dimension in the areas mentioned above, while CEREC exhibited higher precision. Molar and premolar areas emerged as the regions with the greatest discrepancies, both in excess and deficiency, compared to the alginate impression. This difference in dimensions was, however, statistically insignificant overall. 3Shape Trios exhibited the shortest scan times, indicating higher efficiency. Among the intraoral scanners, Medit recorded the longest scanning duration.

**Conclusion.:**

Accepting the null hypothesis, the scans obtained using all three scanners were comparable with statistically insignificant differences in the measurements. The three scanners differed in the total scan time taken, with the Medit scanner requiring the longest scan time and the 3Shape TRIOS 3 scanner demonstrating the shortest scan duration.

## Introduction

 Since the advent of the new millennium, orthodontics has witnessed an integration of popular technologies capable of intricate decision-making, emulating human intelligence in machines, and simulating human actions.^1^ In digital orthodontics, one of the latest innovations is using intraoral scanners, which are chairside devices as alternatives to conventional impression materials, to capture patients’ dentition.^2-5^ This digital transition offers multiple benefits, such as minimized storage demands, rapid access to 3D diagnostic insights, and streamlined digital data transmission for effective communication with both professionals and patients.^6-10^ Moreover, the realm of digital dental models has paved the way for the development of virtual setups, enhancing treatment planning and the production of tailored removable and fixed appliances.^7-11^ The prominence of intraoral scanners is on the rise, with frequent introductions of new devices. Kravitz et al,^10^ in an up-to-date assessment of intraoral digital scanners, proposed that these devices are poised to replace conventional alginate impressions.

 The orthodontic treatment planning relies on a set of study models, photographs, radiographs, and clinical assessments. Traditional impressions stand as the established benchmark. A novel concept of intraoral impressions emerged in 1973 with the introduction of intraoral scanning technology. Subsequently, Sirona Dentsply pioneered a chairside scanning system centered around CAD/CAM technology (known as CEREC).

 Intraoral scanners (IOS), utilizing laser emission, stand as potent optical impression tools, capturing the dimensions and shapes of dental arches. The data is acquired through high-resolution cameras and processed using sophisticated software to generate a polygonal mesh representation, thus forming a “cloud of points.” Further processing yields the final 3D model. Employing non-invasive optical techniques like confocal microscopy, light triangulation, and active wavefront sampling, these scanners do not physically contact the scanned object. A combination of these technologies is used to minimize noise during intraoral scanning and counter distortions arising from saliva. The resulting STL files expedite communication with colleagues and technicians, eliminating the discomfort linked with conventional impressions. However, the alignment between the accuracy of intraoral scanners and traditional impressions remains to be established due to limited research material.

 The creation of virtual setups from optical impressions opens doors to manufacturing a variety of customized orthodontic appliances, such as expanders, aligners, mini-screw assisted devices, and lingual orthodontics. Precision in fabrication is essential to tailor these appliances for individual patients.

 Given the lack of comparative research encompassing the three intraoral scanners, the present in vivo study aims to fill this void. The study’s primary objective is to assess the precision of full-arch digital impressions achieved through different intraoral scanning methods regarding trueness while employing the ideality of conventional impression techniques as a benchmark. The null hypothesis posits that no statistically significant differences exist in the trueness mean between diverse digital impression systems and the traditional impression technique.

## Methods

 This research was conducted within the academic environment of Saveetha Dental College and Hospitals, Chennai. Approval for ethical considerations was granted by the University, and the Ethics Committee identification was issued by the review board under the reference IHEC/SDC/ORTHO-2107/22/171.

**Figure 1:**
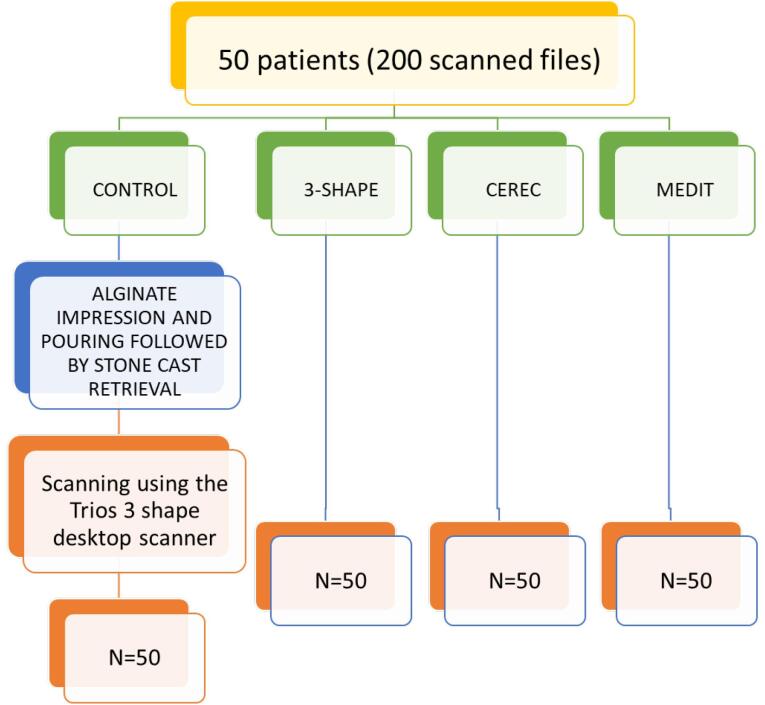
Flowchart summarizing the division of groups

 Sample size determination followed the methodology of Federica Pellitteri et al.’s previous investigation and was facilitated by G power 3.0.10 software.^12^ A power value (P) of 95% was ascertained, leading to a sample size (N) of 50 participants. This encompassed three scanner groups, each comprising 50 files, in addition to a control group, culminating in a total of 200 scanned files. Fifty consecutive subjects (23 males and 27 females) aged 15‒45 were enrolled in the study from January 12 to April 12, 2022. Inclusion criteria mandated complete natural permanent dentition (excluding non-erupted or extracted third molars), the absence of prosthetic or amalgam restorations, and the non-utilization of orthodontic appliances (Figure 1).

**Figure 2:**
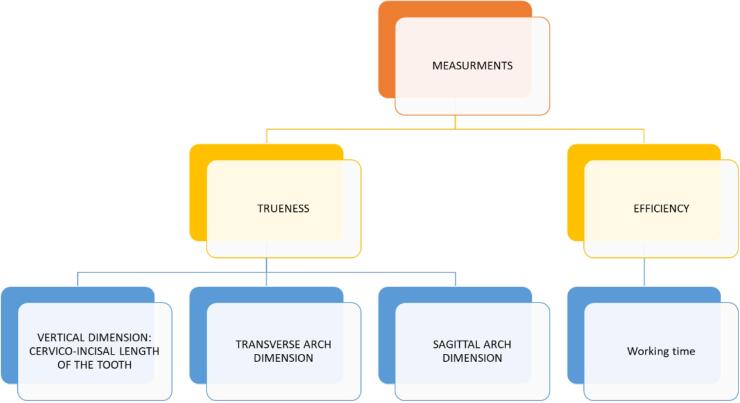
Flowchart summarizing the measurements made

 The clinical procedures were executed by a consistent operator (NB) and subsequently verified by the supervisor (SMP). For the control group, a single-step procedure involved creating an alginate impression that was then cast in dental stone (type IV gypsum). The derived stone casts were subsequently subjected to scanning using 3Shape Trios (3Shape, Copenhagen, Denmark). Subsequently, the same operator proceeded to utilize three distinct intraoral scanners to capture complete arch dentitions of the subjects: Medit (i500), CEREC Primescan (Software 4.6.1, Cerec Primescan®, Dentsply Sirona, Germany), and 3Shape Trios (Software 1.18.2.6, Trios 3®, 3-Shape, Copenhagen, Denmark). The scanning sequence followed precise guidelines outlined in Anh and colleagues’ literature, commencing with the occlusal-palatal facets of the right second molar in the maxilla, progressing toward the opposing side of the arch while consistently encompassing two surfaces, and concluding by revisiting the buccal side.^13^ Upon completing all scanning activities, the scans were converted into STL (Standard Tessellation Language) format and transferred to 3-shape Orthoanalyzer digital software version 2021.1 and Geomagic Design X software. These software packages have diverse functionalities, enabling clinicians to undertake various measurements^14^ (Figure 2).

 This study encompassed various measurements categorized as follows:

 A) Assessment of vertical dimension by measuring the cervico-incisal length of individual teeth

 B) Examination of the transverse arch dimension - encompassing three measurements, namely intermolar distance (6-6), inter premolar distance (4-4), and intercanine distance (3-3)

 C) Analysis of the sagittal arch dimension, performed by measuring the arch length, which involved measuring the line connecting the central incisors’ edges to the intermolar line. Each scan was overlaid onto the scanned stone cast within the software.

 D) To compare the efficiency of digital and traditional workflows, the time taken to complete tasks in minutes was utilized. For intraoral scanners (IOS), the effective work time was calculated by summing the total scan time, software refinement processes, and the time taken to export the STL file. Regarding conventional impressions, the manufacturer-provided times for material mixing, setting, and cast pouring into dental stone were combined. Measurements were made on the virtual model derived from scanning the stone cast, which served as the reference standard.

 The subsequent phase involved duplicating the identical measurements on the reference data for all 50 subjects. A supervisor verified all these measurements after 14 days. All these measured distances were compared to detect potential statistically significant distinctions. This was vital in determining if the distances established between points in the reference data and corresponding points in the three measured data sets were influenced not solely by point placement and operator influence but crucially by the distortion (trueness error) introduced by the employed scanner. Additionally, the transverse dimensions of the arch and the inter-point distances within individual teeth were gauged.

**Figure 3:**
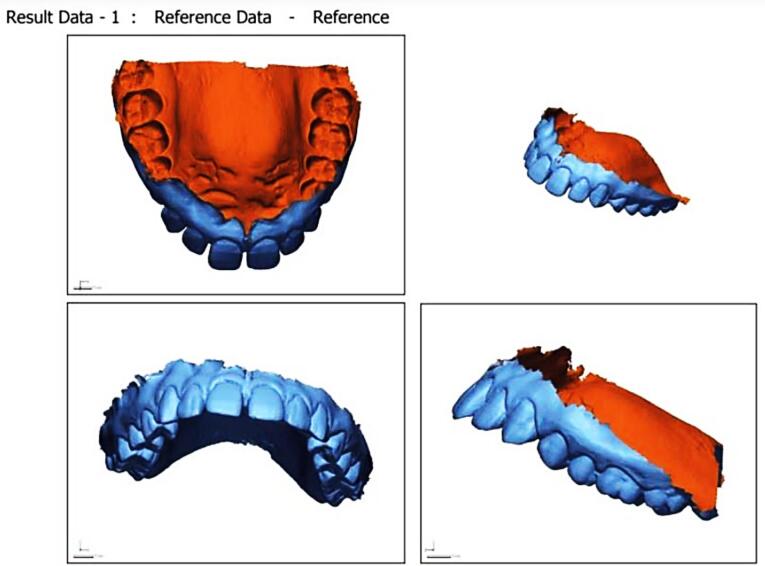
Scan superimposition using the Geomagic software

**Figure 4:**
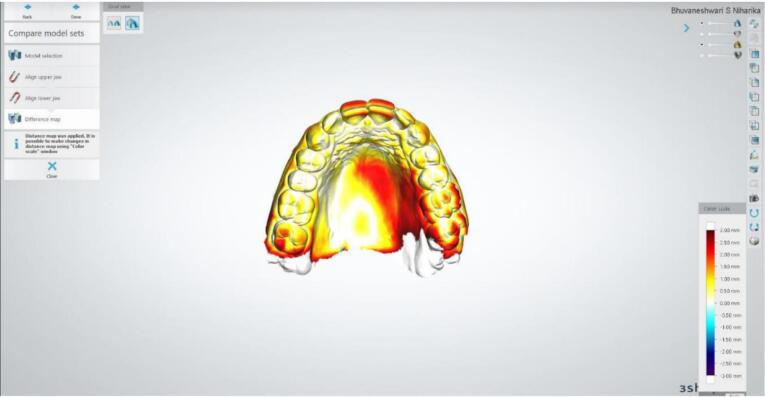
Superimposition of the scan on the ideal model using the Orthoanalyser software

**Figure 5:**
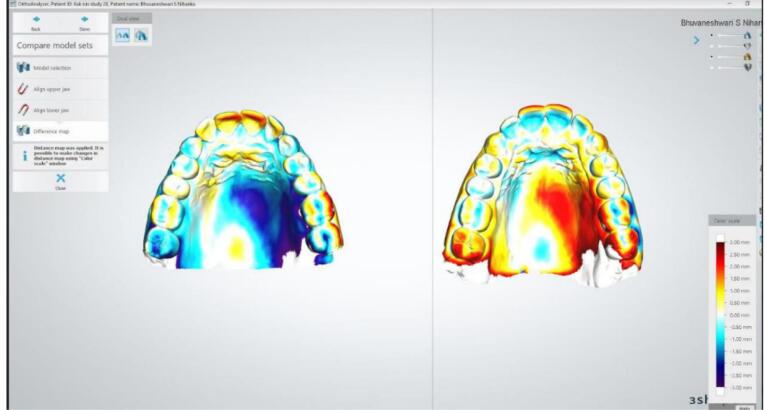
Superimposition of the scans using the Orthoanalyser software

 Subsequently, the alignment of the STL files was carried out using both the 3Shape Orthoanalyser software and the Geomagic Qualify software. The alignment process was executed by Geomagic using Semiautomatic best-fit registration software (S-BF), while the 3Shape Orthoanalyser employed Interactive surface-based registration software (I-SB).^15^ Post-alignment, the model edges were adjusted through digital cutting tools to achieve congruent boundaries between the models. Geomagic Qualify software then computed the maximum and mean distances (including positive and negative differences) as well as the standard deviation amid the “capturing points” of the two digital models (Figure 3). The values from the 3Shape Ortho analyzer were visually represented using a “color map” depicting various colors to determine distances between the models. The threshold for generating this color map was set at 0.25 mm (Figures 4 and 5).

###  Statistical analysis

 The data were organized using Microsoft Excel© and then transferred to SPSS 23 (IBM) for statistical analysis. Data normality was analyzed using the Kolmogorov-Smirnov test, resulting in a parametric outcome. The descriptive statistics (Table 1) were performed, followed by one-way ANOVA (Table 2) to measure the statistical significance, followed by post hoc Tukey tests (α = 0.05) to measure the difference between the groups (Table 3).

**Table 1:** Descriptive statistics

**Groups**		**N**	**Mean**	**SD**
Inter molar distance (6-6)	Group 1 (Model scan)	N = 50	40.66	1.294
	Group 2 (Medit scan)	N = 50	38.06	1.169
	Group 3 (3Shape Trios)	N = 50	39.71	1.078
	Group 4 (CEREC)	N = 50	40.55	1.282
		Total = 200	39.74	0.248
Inter premolar distance (4-4)	Group 1 (Model scan)	N = 50	23.87	0.90
	Group 2 (Medit scan)	N = 50	21.09	1.35
	Group 3 (3Shape Trios)	N = 50	22.62	1.17
	Group 4 (CEREC)	N = 50	23.66	1.02
		Total = 200	22.81	1.55
Inter canine distance (3-3)	Group 1 (Model scan)	N = 50	23.26	2.1
	Group 2 (Medit scan)	N = 50	21.98	3.6
	Group 3 (3Shape Trios)	N = 50	22.34	2.3
	Group 4 (CEREC)	N = 50	23.66	2.1
		Total = 200	22.81	2.6
Sagittal distance	Group 1 (Model scan)	N = 50	43.52	3.4
	Group 2 (Medit scan)	N = 50	41.32	3.3
	Group 3 (3Shape Trios)	N = 50	42.2	3.3
	Group 4 (CEREC)	N = 50	43.3	3.6
		Total = 200	42.6	3.4
Cervicoincisal length	Group 1 (Model scan)	N = 50	9.45	0.99
	Group 2 (Medit scan)	N = 50	8.73	0.64
	Group 3 (3Shape Trios)	N = 50	9.03	0.84
	Group 4 (CEREC)	N = 50	9.41	1.22
		Total = 200	9.1	0.96

**Table 2:** One-way ANOVA

**Groups**	**F**	***P*** ** value**
Intermolar distance (6‒6)	9.8	0.00
Interpremolar distance (4‒4)	12.69	0.00
Intercanine distance (3‒3)	0.549	0.652
Sagittal distance	0.891	0.455
Cervicoincisal length	1.283	0.295

**Table 3:** Post hoc Tukey tests

**Dependent Variable**	**Group**	**Group**	**Mean Difference**	**Sig.**
Intermolar distance (6‒6)	1	2	2.6	0.00
		3	0.95	0.30
		4	0.11	0.99
	2	1	-2.6	0.00
		3	-1.6	0.02
		4	-2.4	0.00
	3	1	-0.9	0.30
		2	1.6	0.02
		4	-0.8	0.41
	4	1	-0.11	0.99
		2	2.4	0.00
		3	0.8	0.41
Interpremolar distance (4‒4)	1	2	2.7	0.00
		3	1.2	0.08
		4	0.21	0.97
	2	1	-2.7	0.00
		3	-1.5	0.022
		4	-2.5	0.00
	3	1	-1.2	0.80
		2	1.5	0.02
		4	-1.0	0.18
	4	1	-0.21	0.97
		2	2.5	0.00
		3	1.04	0.18
Intercanine distance (3‒3)	1	2	1.28	0.711
		3	0.92	0.86
		4	0.09	1.00
	2	1	-1.2	0.71
		3	0.92	0.99
		4	0.09	0.75
	3	1	-0.9	0.86
		2	0.36	0.99
		4	-0.83	0.89
	4	1	-0.09	1.00
		2	1.19	0.75
		3	0.83	0.89
Sagittal distance	1	2	2.20	0.49
		3	1.29	0.83
		4	0.18	0.99
	2	1	-2.2	0.49
		3	-0.91	0.93
		4	-2.02	0.56
	3	1	-1.2	0.83
		2	0.91	0.93
		4	-1.1	0.88
	4	1	-0.18	0.99
		2	2.02	0.56
		3	1.11	0.88
Cervicoincisal length	1	2	0.72	0.34
		3	0.42	0.75
		4	0.04	1.00
	2	1	-0.72	-1.86
		3	-0.30	-1.44
		4	-0.68	-1.82
	3	1	-0.42	0.75
		2	0.30	0.89
		4	-0.38	0.80
	4	1	-0.04	1.00
		2	0.68	0.39
		3	0.38	0.80

Figure 3 is a histogram graph depicting that the mean value for the intermolar distance (6-6) was the highest in group 1- the measurement made on the scanned model, closely followed by that of group 4, the measurement made using CEREC prime scan. The mean values for the transverse dimensions of interpremolar and intercanine distances are also highest in group 1, closely followed by group 4.

 The same is true for the sagittal dimension and the cervicoincisal length of the teeth. The lowest mean value for all the assessed parameters is depicted by group 2, which has the maximum variation from the values shown in group 1 (the control).

## Results

 The results were as follows:

###  A. Assessment of individual tooth dimensions (the vertical dimension)

 The CEREC prime scan yielded the most precise results with a mean difference of only 0.04 mm and a statistically insignificant difference from group 1 (*P* < 0.05). In contrast, the Medit scan exhibited the least accuracy. Nevertheless, the differences in cervicoincisal lengths of individual teeth among the scans displayed clinical and statistical insignificance when compared within groups and with the measurements derived from the stone cast scan.

###  B. Evaluation of transverse dimension

 The CEREC prime scan demonstrated a reduction of 0.11 mm in intermolar width compared to the original model; however, this difference was not significant (*P* > 0.05). Conversely, the Medit scan displayed a more substantial reduction of 2.6 mm in intermolar width, which was statistically significant (*P* < 0.05).

 The CEREC prime scan demonstrated a difference of 0.21 mm in the interpremolar width compared to the original model; however, this difference was not significant (*P* > 0.05). Conversely, the Medit scan exhibited a more substantial difference of 2.7 mm in the interpremolar width, which was statistically significant (*P* < 0.05).

 The CEREC prime scan had a statistically insignificant mean difference of 0.09 mm in the intercanine width (*P* > 0.05), whereas the 3Shape TRIOS 3 scan also revealed a statistically insignificant difference (*P* > 0.05) of 0.92 mm.

**Figure 6:**
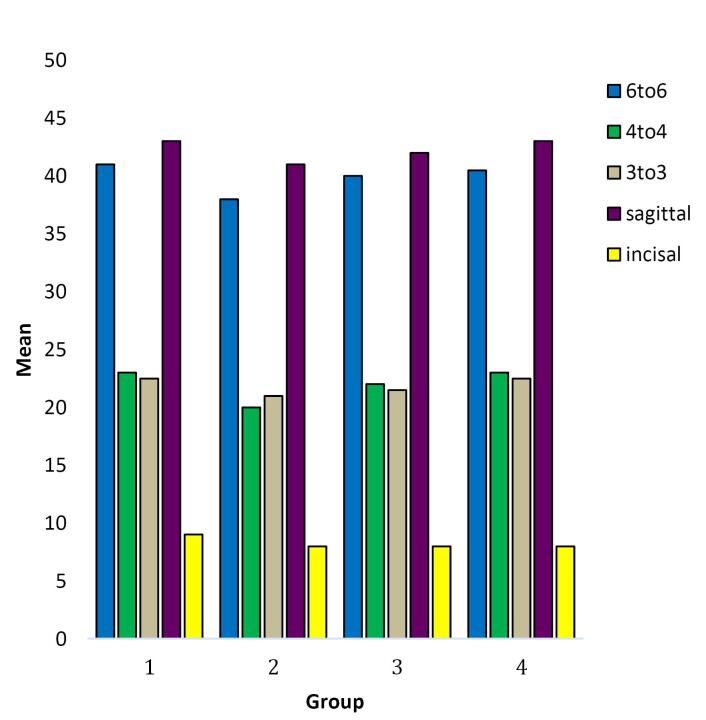
Graph depicting mean outcome variables

 Notably, the Medit scans exhibited the most significant dissimilarity in measurements of the intercanine width compared to group 1; however, this mean difference was not significant (*P* > 0.05) (Figure 6).

###  C. Examination of the sagittal dimension

 Comparing the original model scan of group 1 with the other three groups revealed a mean difference, which was not statistically significant (*P* > 0.05).

###  D. Time taken

 The Medit scanner recorded the lengthiest total scan time at 8 minutes and 3 seconds.

 In contrast, the 3Shape TRIOS 3 scanner registered the shortest total scan time at 3 minutes and 8 seconds.

 The superimposition of the digital models was used to evaluate the mean distances and the standard deviations between the models. The results of the descriptive statistical analyses of the differences between the superimposition of the three digital models are presented in Table 4.

**Table 4:** Descriptive statistical analysis of the differences between the superimposition of the three digital models

**Parameter**	**Minimum**	**Maximum**	**Mean**	**SD**
Upper				
Mean deviation	-0.07	0.04	0.01	0.02
Mean positive difference	0.03	0.18	0.08	0.04
Mean negative difference	-0.20	-0.03	-0.07	0.05
Standard deviation	0.05	0.24	0.1	0.05
Lower				
Mean deviation	0.00	0.08	0.03	0.02
Mean positive deviation	0.03	0.25	0.07	0.04
Mean negative deviation	-0.41	-0.03	-0.06	0.07
Standard deviation	0.05	0.70	0.12	0.12

## Discussion

 In recent years, orthodontic scanner use has grown, offering diverse applications such as full-arch scanning, indirect bonding, fabricating orthodontic appliances, and precise measurements for orthodontists. These applications are crucial for comprehensive planning and model analysis, aiding successful orthodontic treatment and achieving balanced occlusion. Among various methodologies, Bolton’s analysis remains prominent in clinical orthodontic practice for identifying tooth size discrepancies.^16^ Traditionally, clinicians employ alginate impressions to obtain occlusal condition stone models, measuring tooth widths manually using calipers for Bolton’s analysis.^17^ Although calipers are regarded as the gold standard for tooth width analysis, they have limitations such as wear, distortion, and storage issues. Consequently, digital models emerged as a solution to mitigate these challenges in the late 1990s.

 The assessment of intraoral scanner accuracy has drawn a great deal of study interest in digital dentistry. The comparison of several intraoral scanners has been the focus of earlier studies in the literature,^18,19^ which aimed to clarify the accuracy and dependability of each one. To evaluate the performance of the scanners, researchers have used some approaches, including scanning standardized objects, dental imprints, and even patients’ oral cavities. Most frequently, trueness and precision have been considered in this research field, while some have also looked into the impact of scanning techniques, scan duration, and the impact of various dental materials on scanner accuracy. The combined results from these studies have shed light on the advantages and disadvantages of intraoral scanning technologies and served as a foundation for the current manuscript, which aims to deepen our knowledge of the changing intraoral scanner market and its application in contemporary dentistry.

 In a similar study by Pellitteri et al,^12^ the accuracy, in terms of trueness, was measured for three intraoral scanners, namely 3Shape Trios, Carestream CS3600, and CEREC Omnicam, and was compared to the stone casts scanned using the desktop scanners R500 3 shape. This study concluded that the Carestream CS 3600 performed better in terms of inter-arch diameter performance when compared to PVS impressions. At the same time, the 3Shape Trios was discovered to be the most precise single-tooth scanner. The 3D and 2D analyses revealed a trend of increased impression distortion in the molar region compared to the traditional one. The findings of this study are partially consistent with the findings of the current study, which also demonstrated a reduction in the intermolar distance in general. However, this decrease was not statistically significant for the CEREC Primescan but was statistically significant for the Medit i500.

 An in-vitro study by Renne et al^20^ compared and evaluated the accuracy and precision of six intraoral scanners and one laboratory scanner in both sextant and complete-arch scenarios. Moreover, scanning speed was assessed and linked to accuracy and precision. Of the intraoral scanners, the 3Shape Trios was found to have the poorest trueness and precision for sextant scanning (*P* < 0.001), while the PlanScan was found to have the best.

 In another study, Kwon et al^21^ concluded that concerning trueness, errors in the intermolar dimension and the distance from the canine to the contralateral molar were greater with Omnicam than with the other scanners, namely i500, CS3600, iTero, and Trios 3. Concerning precision, the error in the linear distance from the canine to the molar in the same quadrant was greater with Omnicam and CS3600 than with the other scanners. However, the results of our current investigation differ from the results of the study by Kwon et al^21^ because the largest error was observed in the current study in connection to scans taken using the Medit i500 scanner.

 A systematic review by Goracci et al^22^ showed that, up to 2016, only a limited number of published studies, totaling eight, had engaged in intraoral complete-arch scanning. Among these, only four studies presented comprehensive data concerning the accuracy, consistency, and reproducibility of digital measurements. Notably, only two of the various intraoral scanners available on the market, namely Lava COS and iTero, had undergone clinical evaluation in these investigations.

 In another systematic review by Jedliński et al,^23^ sixteen studies were included, in which four RCTs and 12 case‒control studies were included. Different scanners were analyzed: 3Shape Trios (3Shape, Copenhagen, Denmark) in 12 studies, iTero Element (Aligntech, San Jose, CA, USA) in four studies, Carestream 3600 (Carestream, Rochester, NY, USA) in four studies, Ortho InSight 3D* (Motion View Software, LLC, Chattanooga, TN, USA) in three studies, Lavacos (3M, Maplewood, MN, USA) in two studies. This systematic review concluded that the scanners of the same generation from different manufacturers have almost identical accuracy.

 In light of the ambiguity surrounding the accuracy and reliability of intraoral scanners currently available on the market, our study was undertaken to provide valuable insights into this critical area of research. The lack of a comprehensive understanding of which intraoral scanner offers the highest precision and consistency poses a substantial challenge for dental practitioners and researchers. Therefore, our investigation addressed this knowledge gap by rigorously assessing the performance of the three most widely used intraoral scanners in an orthodontic practice, which have not previously been studied and compared together, namely CEREC Primescan, Medit, and 3Shape Trios intraoral scanners, considering various aspects of accuracy, trueness, repeatability, and scanning time. Dentsply Sirona’s CEREC Primescan Medit i500 and 3Shape Trios use optical triangulation and confocal laser scanning technologies, respectively, resulting in fast and highly accurate scanners. This study delved into and compared the accuracy of the three intraoral scanners across three dimensions: transverse, sagittal, and vertical. Additionally, it examined the efficiency in terms of the time required for complete scans of the maxillary occlusal, mandibular occlusal, and bite scans. By doing so, we aim to provide practitioners and the dental community with a more informed perspective on the efficacy of these technologies, ultimately enhancing the quality of digital dentistry applications.

 The results of the present study were partially consistent with previous studies on a similar topic. The results of our study, focusing on various dimensions and aspects of intraoral scanning, provide important insights into the performance of different scanners. In assessing individual tooth dimensions, specifically the vertical dimension, the CEREC Prime scan demonstrated the highest precision, with a mean difference of only 0.04 mm, while the Medit scan exhibited the least accuracy. Nonetheless, the clinical and statistical insignificance of differences in cervicoincisal lengths between the scans, when compared within groups and with measurements from the stone cast scan, indicates the overall reliability of these scanners for this dimension. In evaluating transverse dimensions, the CEREC Prime scan displayed a statistically insignificant reduction in intermolar and interpremolar widths, whereas the Medit scan showed a statistically significant reduction. For intercanine width, both the CEREC Prime and 3Shape TRIOS 3 scans presented statistically insignificant differences, while the Medit scan exhibited statistically negligible variations. In examining sagittal dimensions, the comparisons between the original model scan of group 1 and the other three groups revealed statistically insignificant differences, reinforcing the scanners’ overall consistency in this dimension.

 Furthermore, our study found that the dimensions of the cervicoincisal length of the teeth among the groups displayed statistically insignificant differences. Regarding scan time, the Medit scanner required the longest total scan time at 8 minutes and 3 seconds, whereas the 3Shape TRIOS 3 scanner was the most time-efficient, with a total scan time of 3 minutes and 8 seconds. These findings collectively emphasize the varying performance characteristics of intraoral scanners and their potential impact on clinical practice and efficiency. The comparison of the scanners is summarized in Table 5.

**Table 5:** Summary of the accuracy of scanners in different dimensions and time

**Parameter**	**CEREC Prime Scan**	**Medit i500**	**3Shape Trios **
Vertical dimension	√	√	√
Transverse dimension	√	×	√
Sagittal dimension	√	√	√
Time taken	Moderate	Maximum	Minimum

 The accuracy of commercially available IOS devices varies, with newer generations exhibiting broader clinical applications. This, coupled with attributes like scanning speed, wand dimensions, and color image capture, shapes the decision to invest in an IOS. These systems can be closed, generating proprietary files, or open, producing files compatible with various CAD software. The former suits less experienced users, while the latter offers enhanced usability. As different scanning technologies emerge, variations in accuracy might emerge. The study’s limitations encompass potential variations due to operator experience and the absence of fixed reference points in software-based measurements, leading to discrepancies between operators. Factors like saliva could also impact scans and introduce discrepancies.

## Conclusion

 The role of technology is progressively gaining significance within the daily practice of orthodontics.^24^ Despite certain limitations, upon a thorough evaluation of the findings from this ongoing in vivo study, it is viable to assert that digital impressions can serve as a viable alternative to the conventional impression technique for making measurements. The null hypothesis is accepted, thereby permitting the assertion that scans derived from either of the three scanners, namely, the CEREC Prime scan, Medit i500, or 3Shape TRIOS 3, do not differ in terms of trueness in either of the three dimensions and show statistically insignificant difference. However, the scanners differ in efficiency, with the Medit scanner requiring the longest scan time, whereas the 3Shape TRIOS 3 scanner demands the shortest scan time.

## Competing Interests

 The authors declare no competing interests.

## Ethical Approval

 This research was conducted within the academic environment of Saveetha Dental College and Hospitals, Chennai. Approval for ethical considerations was granted by the University, and the Ethics Committee identification was issued by the review board under the reference IHEC/SDC/ORTHO-2107/22/171.

## Funding

 This work received no funding.
